# Modeling adaptation of carbon use efficiency in microbial communities

**DOI:** 10.3389/fmicb.2014.00571

**Published:** 2014-10-28

**Authors:** Steven D. Allison

**Affiliations:** ^1^Department of Ecology and Evolutionary Biology, University of California IrvineIrvine, CA, USA; ^2^Department of Earth System Science, University of California IrvineIrvine, CA, USA

**Keywords:** bacteria, climate change, fungi, rate-yield tradeoff, soil carbon, temperature, theoretical model

## Abstract

In new microbial-biogeochemical models, microbial carbon use efficiency (CUE) is often assumed to decline with increasing temperature. Under this assumption, soil carbon losses under warming are small because microbial biomass declines. Yet there is also empirical evidence that CUE may adapt (i.e., become less sensitive) to warming, thereby mitigating negative effects on microbial biomass. To analyze potential mechanisms of CUE adaptation, I used two theoretical models to implement a tradeoff between microbial uptake rate and CUE. This rate-yield tradeoff is based on thermodynamic principles and suggests that microbes with greater investment in resource acquisition should have lower CUE. Microbial communities or individuals could adapt to warming by reducing investment in enzymes and uptake machinery. Consistent with this idea, a simple analytical model predicted that adaptation can offset 50% of the warming-induced decline in CUE. To assess the ecosystem implications of the rate-yield tradeoff, I quantified CUE adaptation in a spatially-structured simulation model with 100 microbial taxa and 12 soil carbon substrates. This model predicted much lower CUE adaptation, likely due to additional physiological and ecological constraints on microbes. In particular, specific resource acquisition traits are needed to maintain stoichiometric balance, and taxa with high CUE and low enzyme investment rely on low-yield, high-enzyme neighbors to catalyze substrate degradation. In contrast to published microbial models, simulations with greater CUE adaptation also showed greater carbon storage under warming. This pattern occurred because microbial communities with stronger CUE adaptation produced fewer degradative enzymes, despite increases in biomass. Thus, the rate-yield tradeoff prevents CUE adaptation from driving ecosystem carbon loss under climate warming.

## Introduction

Climate warming is expected to alter biogeochemical processes in ecosystems (Davidson and Janssens, 2006; Bardgett et al., 2008). All of these processes are influenced by micro-organisms like bacteria and fungi, and many predictive models now include some form of microbial control over biogeochemistry (Moorhead and Sinsabaugh, 2006; Lawrence et al., 2009; Allison et al., 2010; Wang et al., 2013; Wieder et al., 2013). In contrast to conventional models in which decomposition is strictly a first-order process, the new models couple microbial biomass with substrate pools (Todd-Brown et al., 2012). This change in model structure, first developed in detail by Schimel and Weintraub (2003), results in biogeochemical rates that depend not only on donor pool sizes (e.g., soil carbon) but also on recipient pools—specifically microbial biomass.

The new microbial models are important because they may be more biologically realistic, and their predictions differ from conventional models under environmental change (Allison et al., 2010; Wieder et al., 2013). At the global scale for instance, one of these new microbial models reproduced existing distributions of soil carbon more accurately than conventional models (Wieder et al., 2013). In response to climate warming, this new model showed a wider range of soil carbon outcomes compared to conventional models, with either large losses or small changes predicted depending on microbial physiological parameters.

Due to the dependence of biogeochemical rates on microbial biomass pools, microbial models are sensitive to physiological parameters that regulate microbial dynamics. Initial studies have shown that carbon use efficiency (CUE) is one of these key parameters (Allison et al., 2010; Sinsabaugh et al., 2013; Li et al., 2014). CUE is defined as the fraction of microbial assimilation that is allocated to biosynthetic processes (e.g., growth), with the remainder typically respired (Steinweg et al., 2008; Manzoni et al., 2012). Thus, greater CUE results in more microbial biomass because more of the assimilated substrate remains in cells rather than being respired.

Most of the initial studies with microbial models have included scenarios in which CUE declines with increasing temperature (Allison et al., 2010; Wang et al., 2013; Wieder et al., 2013; Li et al., 2014). This relationship is based on empirical studies that have found reductions in CUE with increasing temperature in the range of 0.009 mg mg^−1^ °C^−1^ to 0.049 mg mg^−1^ °C^−1^ (Devêvre and Horwath, 2000; Van Ginkel et al., 2000; Rivkin and Legendre, 2001; Pietikäinen et al., 2005; Apple et al., 2006; Steinweg et al., 2008), although the relationship is not always consistent (Del Giorgio and Cole, 1998; Dijkstra et al., 2011). Physiologically, CUE should decline with warming if respiratory processes are more temperature sensitive than growth processes (Farmer and Jones, 1976; Mainzer and Hempfling, 1976; Hall and Cotner, 2007). Processes such as protein turnover and cell repair might increase at higher temperatures, though the exact mechanism underlying the CUE-temperature relationship in soils has not been determined (Bradford, 2013).

A recent study from the Harvard Forest warming experiment found evidence that the temperature sensitivity of CUE may decline in response to long-term warming (Frey et al., 2013). There is also some evidence for seasonal changes in microbial community CUE (Tucker et al., 2013). Several mechanisms could lead to this “adaptation” of CUE. Over time, shifts in microbial community composition or substrate use could reduce CUE temperature sensitivity. More efficient microbial taxa might be selected for, or microbes might switch to using substrates that can be assimilated with greater efficiency. Still, if efficiency gains are possible, why aren't they already realized, even at lower temperatures?

Many physiological studies going back decades show that increased efficiency trades off against rates of resource consumption (Pirt, 1965; Pfeiffer et al., 2001; Frank, 2010). Cells with higher consumption rates have lower CUE and vice versa. Rapid resource consumption requires more cellular machinery such as uptake proteins, ribosomes, and metabolic enzymes that increase respiratory costs. Increasing CUE by reducing these costs therefore comes at the expense of a lower resource acquisition rate.

For free-living, heterotrophic microbes, fast growth requires metabolic machinery to degrade and take up complex resources from the environment (Koch, 1985, 1997). This machinery takes the form of extracellular enzymes and uptake proteins that target energy- and nutrient-containing molecules. Cells that have a greater enzymatic capacity should process complex resources more rapidly, but should also incur relatively greater respiratory costs that reduce CUE. If these respiratory costs increase faster than the benefits of enzyme production as temperatures rise, then microbes might be expected to reduce enzyme investment with warming.

The goal of this study was to model the consequences of the rate-yield tradeoff for carbon cycling responses to temperature increase. Although there is some empirical evidence that community-level CUE adapts to temperature (Frey et al., 2013), the mechanisms, implications, and generality of this response are poorly known. Theoretical models offer a tractable means of identifying potential mechanisms and generating testable hypotheses.

I analyzed the rate-yield tradeoff with a simple analytical model and with the DEMENT model (Allison, 2012). DEMENT includes substrate feedbacks and represents microbial diversity by assigning physiological traits to virtual taxa that compete in a spatially-structured environment. Ecosystem process rates (i.e., decomposition) and community properties (i.e., functional diversity) emerge from the model dynamics (Follows et al., 2007). The analytical model makes clear, idealized predictions about the potential for microbial adaptation to temperature, whereas DEMENT imposes additional constraints through substrate and microbial interactions. I tested whether the potential for CUE change under warming was similar for the two theoretical systems and analyzed mechanisms underlying the differences between models.

## Materials and methods

### Analytical model

The analytical model was constructed to analyze the potential for CUE adaptation in a spatially and taxonomically homogeneous microbial community. The model objective was to find the CUE that maximizes microbial growth (*G*), which is assumed to equal the uptake rate (*U*) times CUE (ε):

(1) G=U∗ε

where *U* follows the Arrhenius relationship with temperature (*T*; °C):

(2) $$U=U_{ref}*\exp\left[\left(-\frac{E_{a}}{R}\right)\left(\frac{1}{T+273}-\frac{1}{T_{ref}+273}\right)\right]$$

*U_ref_* is the uptake rate at the reference temperature (*T_ref_* = 20°C), *E_a_* is the activation energy for uptake, and *R* is the ideal gas constant (8.314 J mol^−1^ K^−1^). CUE is assumed to vary linearly with *T*, where ε_*int*_ is intrinsic CUE at 20°C and *m_T_* is the temperature sensitivity of CUE:

(3) $$\varepsilon =\varepsilon_{int}+m_{T}\;*\;(T-T_{ref})$$

To implement the growth-yield tradeoff, ε_*int*_ is assumed to vary linearly with reference uptake rate with intercept ε_0_ and slope *m_U_*.

(4) $$\varepsilon_{int}=\varepsilon_{0}+m_{U}\;*\;U_{ref}$$

Substituting Equations (2–4) into Equation (1), differentiating *G* with respect to *U_ref_*, and solving the differential equation for *U_ref_* yields:

(5) $$U_{ref}=-\frac{\varepsilon_{0}+m_{T}\;*\;(T-T_{ref})}{2m_{U}}$$

Substituting Equation (5) into Equation (4) yields:

(6) $$\varepsilon_{int}=\frac{\varepsilon_{0}-m_{T}\;*\;(T-T_{ref})}{2}$$

Thus if higher uptake rates trade off against lower CUE, the model predicts that intrinsic CUE must increase linearly as temperature increases in order to maximize growth. Greater intrinsic CUE helps offset the decline in realized CUE with increasing temperature. However, greater intrinsic CUE comes at the cost of reducing uptake potential, *U_ref_*. The analytical model was analyzed with parameters given in Table 1 but with *m_U_* equal to −0.2 and −0.4 to represent high and low tradeoff scenarios, respectively.

**Table 1:** **Values and units for model parameters**.

**Variable**	**Value**	**Units**	**Interpretation (with reference if available)**
*t*	5000	Day	Number of iterations
*N_E_*	50		Number of enzymes in community
*N_S_*	12		Number of substrates
*N_U_*	14		Number of uptake transporters
*N_B_*	100		Number of taxa
*E_a_*	35	kJ mol^−1^	Activation energy for uptake
*E_aK_*	20	kJ mol^−1^	Activation energy for *K_m_* (German et al., 2012)
*K_mESlope_*	10	mg enzyme day cm^−3^	Slope for *K_m_* – *V_E_* relationship
*K_mEInt_*	0	mg cm^−3^	Intercept for enzyme *K_m_* – *V_E_* relationship
*K_mUSlope_*	0.2	mg biomass day cm^−3^	Slope for *K_m_* – *V_U_* relationship
*K_mUInt_*	0	mg cm^−3^	Intercept for uptake *K_m_* – *V_U_* relationship
*V_E_*	100	mg substrate mg^−1^ enzyme day^−1^	*V_max_* for enzymes
*V_U_*	5	mg substrate mg^−1^ biomass day^−1^	*V_max_* for uptake
λ_*Slope*_	−0.8		Fractional change in cellulose decay per unit lignocellulose index
*E_S_*	1		Minimum number of enzymes capable of degrading each substrate
*U_M_*	1		Minimum number of uptake transporters capable of taking up each monomer
*E_max_*	40		Maximum number of enzymes a taxon may produce
θ	1		Coefficient determining strength of specificity-efficiency tradeoff
ε_0_	0.5	mg mg^−1^	Intercept for C use efficiency function (Thiet et al., 2006)
*m_T_*	−0.016	mg mg^−1°^C^−1^	C use efficiency temperature sensitivity (Allison et al., 2010)
*m_E_*	−0.1, −0.2	mg mg^−1^	C use efficiency change with enzyme investment
*m_U_*	−0.1, −0.2	mg mg^−1^	C use efficiency change with uptake investment
*Z_EC_*	5×10^−5^	mg mg^−1^	Per enzyme C cost as a fraction of uptake rate
β_*EC*_	5×10^−5^	mg mg^−1^ day^−1^	Per enzyme C cost as a fraction of biomass
*Z_EN_*	0.3	mg mg^−1^	Per enzyme N cost as a fraction of C cost (Sterner and Elser, 2002)
*L*	0.1	day^−1^	Leaching rate
τ_*E*_	0.04	day^−1^	Enzyme turnover rate (Allison, 2006)
τ_*B*_	0.02	day^−1^	Bacterial turnover rate (Schimel and Weintraub, 2003)
τ_*F*_	0.01	day^−1^	Fungal turnover rate (Rousk and Bååth, 2007)
*F_MS_*	0.045	mg mg^−1^	Initial monomer present as a fraction of initial substrate
*D_B_*	0.1		Initial bacterial cell density per lattice point
*D_F_*	0.004		Initial fungal cell density per lattice point
*C_B_*	0.825	mg mg^−1^	Bacterial C fraction (Sterner and Elser, 2002)
*N_B_*	0.160	mg mg^−1^	Bacterial N fraction (Sterner and Elser, 2002)
*P_B_*	0.015	mg mg^−1^	Bacterial P fraction (Sterner and Elser, 2002)
*C_F_*	0.900	mg mg^−1^	Fungal C fraction (Sterner and Elser, 2002)
*N_F_*	0.090	mg mg^−1^	Fungal N fraction (Sterner and Elser, 2002)
*P_F_*	0.010	mg mg^−1^	Fungal P fraction (Sterner and Elser, 2002)
*C_l_*	0.090	mg mg^−1^	Tolerance on C fraction
*N_l_*	0.040	mg mg^−1^	Tolerance on N fraction
*P_l_*	0.005	mg mg^−1^	Tolerance on P fraction
*C_min_*	0.086	mg cm^−3^	Threshold C concentration for cell death
*N_min_*	0.012	mg cm^−3^	Threshold N concentration for cell death
*P_min_*	0.002	mg cm^−3^	Threshold P concentration for cell death
*C_Bmax_*	2	mg cm^−3^	C concentration threshold for bacterial reproduction
*C_Fmax_*	50	mg cm^−3^	C concentration threshold for fungal reproduction
*F_B_*	0.5		Initial biomass fraction of fungi
ρ_*y*_	0.05		Probability of fungi dispersing in *y* direction
δ	1	lattice point	Maximum dispersal distance
*T*	15, 20	°C	Temperature
*x*	100		Lattice length
*y*	100		Lattice width

### Dement model

To represent CUE changes in complex microbial communities, I used the DEMENT simulation model (Allison, 2012) with a modification to represent fungal growth strategies. DEMENT is a spatially-explicit, agent-based model of organic matter decomposition driven by extracellular enzymes. Microbial cells are located on a lattice (100 × 100 points) with wrap-around boundaries, and multiple cells may occupy the same lattice point. The model represents microbial diversity through the random assignment of physiological and enzymatic traits to virtual taxa. Taxa with favorable traits in a given environment increase in abundance. Tradeoffs among traits can be represented as correlations; for example, a taxon that is randomly assigned a high enzyme production rate will be assigned a low CUE.

For this study, I simulated 100 taxa (50% bacteria and 50% fungi initially) that could each produce between 0 and 40 hypothetical extracellular enzymes. Individual enzyme activities were assigned at random, with each enzyme targeting at least 1 of 12 organic substrates. Enzymes degrade substrates into monomers that microbial cells target with uptake proteins. Each taxon was assigned between 1 and 14 uptake proteins that each target at least 1 monomer or inorganic N or P produced through mineralization. Enzymes and uptake proteins were assigned to taxa at random, but taxa were forced to possess uptake proteins for at least 1 organic monomer and for all the monomers released by the extracellular enzymes they were assigned.

Enzyme and uptake kinetics follow the Michaelis-Menten relationship. *V_max_* and *K_m_* values were held constant across different enzymes and across different uptake proteins (Table 1). However, enzymes active against more than one substrate had reduced *V_max_* values under the assumption of a specificity-efficiency tradeoff. Uptake potential was assumed to be proportional to cell mass. Enzyme production has a constitutive component proportional to cell biomass and an inducible component proportional to monomer uptake. Enzyme and uptake *V_max_* and *K_m_* follow the Arrhenius relationship with temperature. Note that the previous version of DEMENT assumed linear temperature of *K_m_*, but I used Arrhenius dependence here to be more consistent with empirical data (German et al., 2012; Stone et al., 2012). DEMENT is stoichiometrically constrained and tracks C, N, and P. Substrate stoichiometry is fixed for each chemical compound, and monomer stoichiometry follows substrate stoichiometry (Table 2). Enzyme stoichiometry is also fixed and constant across enzymes. Microbial biomass stoichiometry is partially homeostatic such that it may vary within limits determined by cell quota ranges for C, N, and P. Following monomer uptake, excess C is respired and excess nutrients are mineralized. Note that excess C respired due to stoichiometric constraints is not counted in calculations of intrinsic CUE.

**Table 2:** **Initial pool sizes, input rates, and activation energies (*E_aS_*) for decay of chemical substrates in the DEMENT model**.

	**Initial pool (mg cm^−3^)**			**Input rate (mg cm^−3^ day^−1^)**		***E_aS_* (kJ mol^−1^)**
	***C***	***N***	***P***	**Substrate**	**Monomer**	
Dead microbe	0	0	0	0	0	37
Dead enzyme	0	0	0	0	0	35
Cellulose	146.89	0	0	0.4024	0.01811	36
Hemicellulose	85.86	0	0	0.2352	0.01058	35
Starch	12.21	0	0	0.0335	0.00151	35
Chitin	5.00	0.8325	0	0.0137	0.00062	37
Lignin	48.51	0.4043	0	0.1329	0.00598	39
Protein 1	10.60	2.0970	0	0.0290	0.00131	35
Protein 2	10.60	2.0970	0	0.0290	0.00131	35
Protein 3	10.60	2.0970	0	0.0290	0.00131	35
Organic P 1	12.48	0	0.4785	0.0342	0.00154	36
Organic P 2	1.82	0.7975	0.4785	0.0050	0.00022	34

*The distribution of chemical substrates is based on Allison et al. (2013). Activation energies are based on McClaugherty and Linkins (1990) and Trasar-Cepeda et al. (2007)*.

Microbial biomass turns over due to starvation or random death. Starvation occurs when one or more nutrient quotas fall below minimum values. Random death is a first-order process with rate constant τ. Enzyme decay is also a first-order process. Dead microbes and decayed enzymes each enter their own substrate pools; the stoichiometry of the dead microbial biomass pool is unconstrained.

To incorporate fungi into DEMENT, I assumed that fungal cell size is larger, dispersal of dividing cells is directional, and that nutrients can be translocated across the entire lattice for fungal taxa. Bacterial dispersal occurs within one lattice point at random directions after the progenitor cell reaches a threshold biomass. This representation generates globular colonies as expected for bacteria. To mimic fungal growth, I increased the minimum cell C for division to 25 times the bacterial value and introduced a directionality parameter which is the total probability of moving either up or down the *y* axis of the lattice. Fungal turnover was assumed to be slower than bacterial turnover by a factor of 2. To implement nutrient translocation, the nutrient quotas for all cells of a given fungal taxon were set to the lattice average at the start of each model iteration, effectively mixing the nutrients across all cells within a fungal taxon.

To implement the rate-yield tradeoff in DEMENT, intrinsic CUE was assumed to decline in proportion to the number of extracellular enzymes and uptake proteins possessed by a taxon. Enzyme investment is expressed as a value *f_E_* between 0 and 1 that represents the fraction of enzymes possessed out of the total possible (40 in this case). Likewise, uptake investment *f_U_* is the fraction of uptake proteins possessed out of the total possible (14 in this case). Intrinsic CUE was expressed as a linear function of these two fractions:

(7) $$\varepsilon_{int}=\varepsilon_{0}+f_{E}\;*\;m_{E}+f_{U}\;*\;m_{U}$$

Note that this relationship is mathematically similar to Equation 4 with *U_ref_* = *f_E_* + *f_U_* and assumes that maintaining more genes for enzymes and uptake results in a higher metabolic burden on the cell. During simulations, this assumption means that taxa with greater investment in enzymes and uptake respire a greater fraction of C upon monomer uptake. There is also a separate biomass cost in terms of C and N associated with enzyme production.

To test for CUE adaptation in DEMENT, I ran paired, 5000-day simulations at 15 and 20°C under a low CUE tradeoff scenario (*m_E_* = *m_U_* = −0.1) and a high CUE tradeoff scenario (*m_E_* = *m_U_* = −0.2). Simulations were initialized with random placement of taxa at an expected mean density of 0.1 bacterial cells or 0.004 fungal cells per lattice point. Because fungal cells are 25 times larger, their initial frequency is lower to hold biomass density constant. I used the same random seed for each pair of 15 and 20°C simulations to hold initial conditions constant and eliminate variation due to different enzyme traits and positioning of taxa on the lattice. Twenty pairs of simulations were run for each scenario. Substrate chemistry reflected litter inputs of southern California grassland (Allison et al., 2013). Initial pools and fluxes are given in Table 2.

Simulation outputs were analyzed by examining the change in intrinsic CUE, microbial biomass, and substrate pools with a 5°C increase in temperature. Community-level intrinsic CUE was calculated as a biomass-weighted average across the 5000-day simulation. Microbial biomass and substrate pool sizes were also averaged across the simulation. Differences among paired simulations were analyzed with one-sample *t*-tests. Relationships among the three output variables were assessed with linear regression.

## Results

### Analytical model

**Figure 1:**
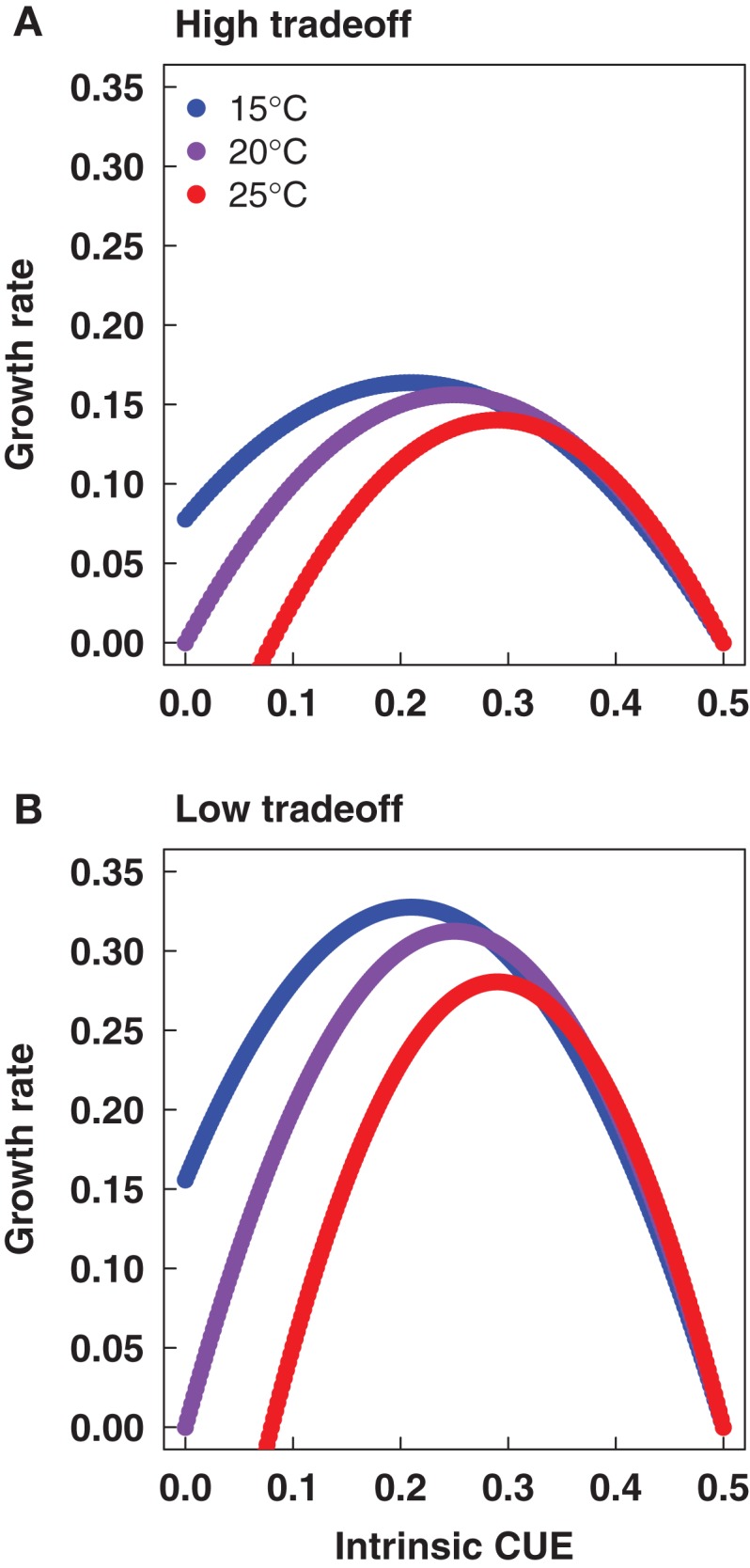
**Relative growth rate as a function of intrinsic carbon use efficiency (CUE) at different temperatures from the analytical model under the (A) high tradeoff (*m_U_* = −0.4) and (B) low tradeoff (*m_U_* = −0.2) scenarios**.

Based on Equation (6), the analytical model predicts a change in intrinsic CUE equivalent to one-half the CUE change forced by temperature increase. This change in intrinsic CUE is associated with a linear decline in reference uptake rate as temperature increases (Figure 1). Thus, greater intrinsic CUE trades off against uptake potential, reflecting the underlying rate vs. yield assumption of the analytical model.

Equation (6) also shows that the magnitude of CUE adaptation is independent of the tradeoff magnitude. Thus, steeper tradeoffs are not expected to increase the potential for adaptation. In contrast, growth rates decline as the tradeoff increases because the effective cost of uptake increases (Figure 1). At the limit of no tradeoff, there is no cost to investing in growth and therefore growth is maximized at infinite uptake investment.

### Dement model

**Figure 2:**
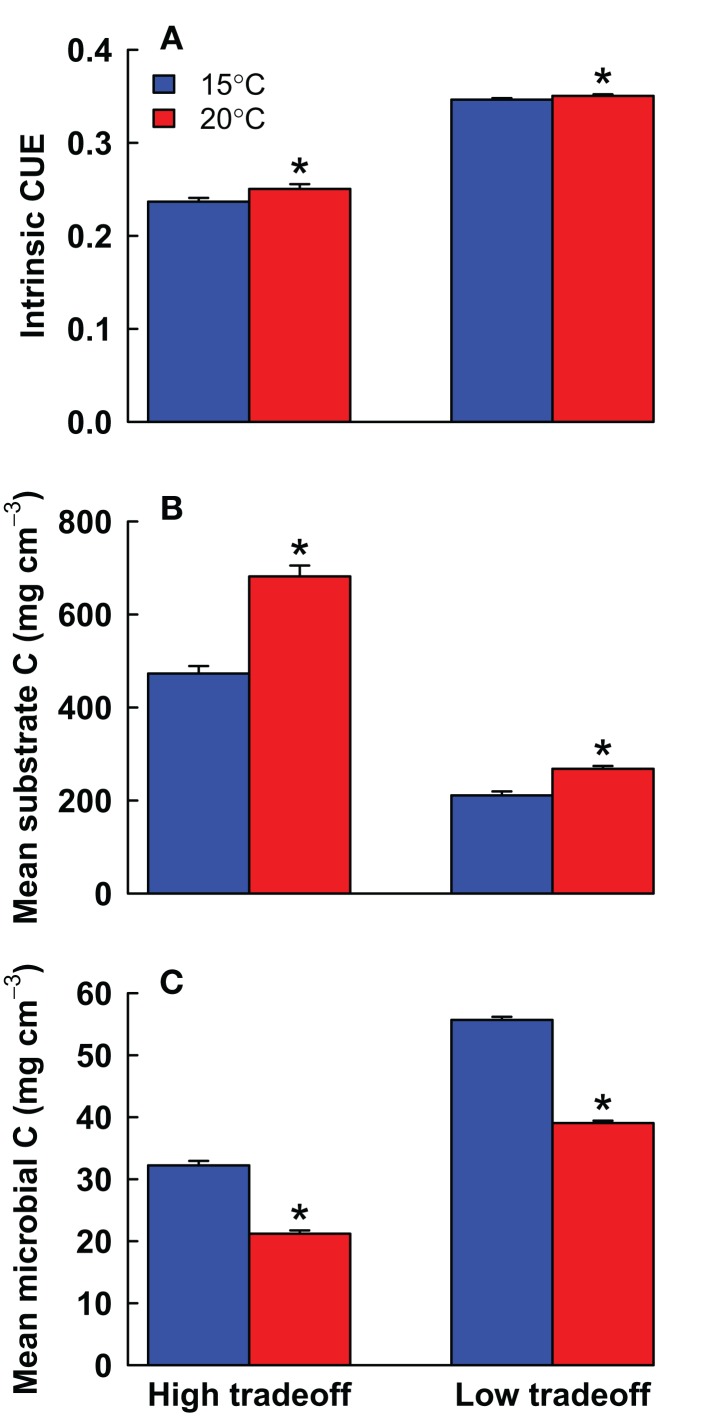
**Mean ± SE (A) intrinsic carbon use efficiency (CUE), (B) total substrate carbon, and (C) total microbial biomass carbon under high and low tradeoff scenarios at 15 vs. 20°C in the DEMENT model**. Significant differences between temperatures are noted with an asterisk (*P* < 0.01, paired *t*-test).

In line with the analytical model, DEMENT showed a significant (*P* < 0.001, *t*-test) increase in intrinsic CUE with warming under the high tradeoff scenario (Figure 2). Yet the magnitude of adaptation was only 0.014 mg mg^−1^ out of the 0.080 mg mg^−1^ of temperature-induced change in CUE. For the low tradeoff scenario, the magnitude of adaptation was even lower, at 0.004 mg mg^−1^. Under the high tradeoff scenario, this level of adaptation corresponds to an average reduction of ~1.4 enzyme genes plus ~0.5 uptake genes, whereas the decline would have to be 4 genes (and 1.4 uptake genes) to match the analytical model. Thus, the taxa that are most abundant under warming had only slightly fewer enzyme genes compared to the dominant taxa under control conditions.

**Figure 3:**
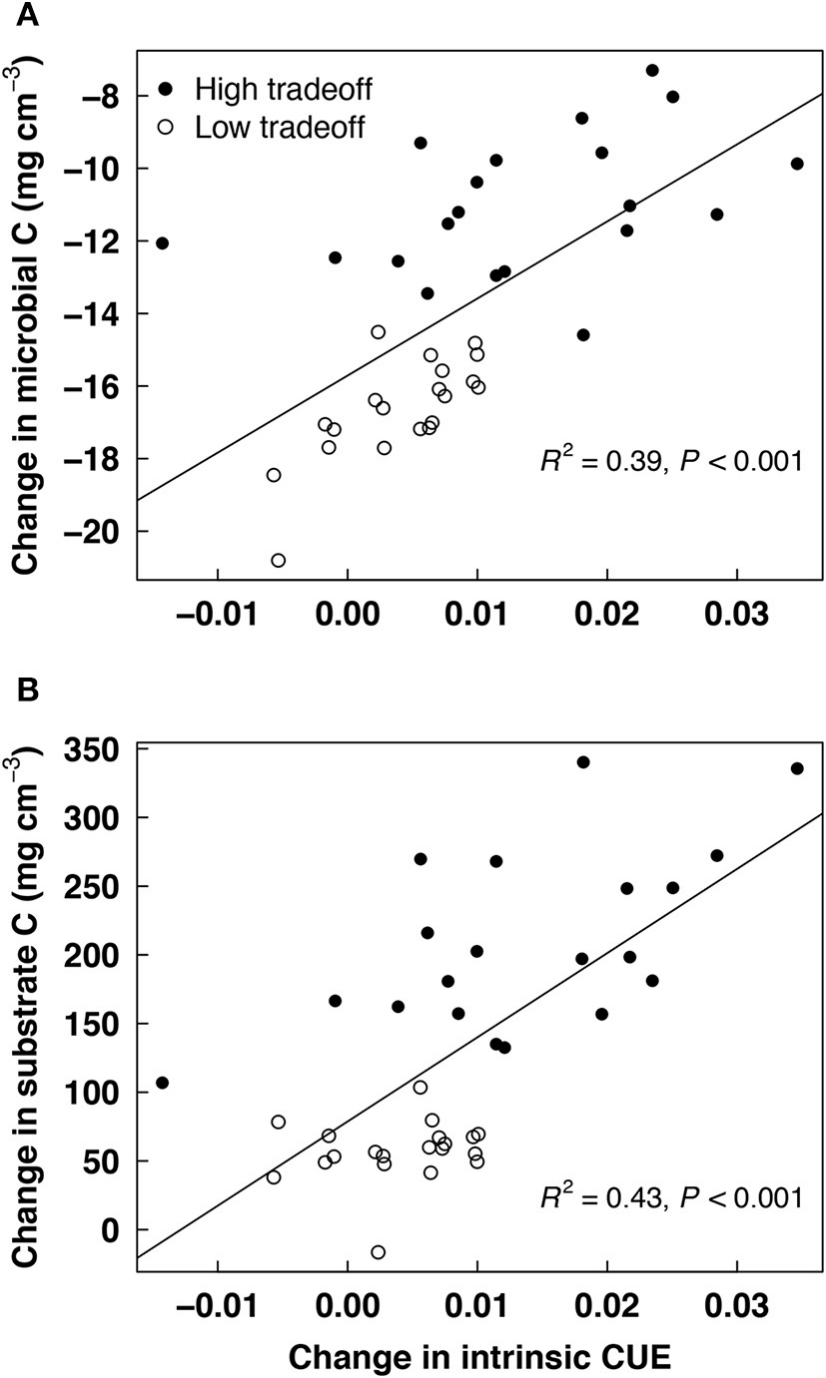
**Relationship between change in intrinsic carbon use efficiency (CUE) with 5°C warming (20°C minus 15°C) and change in (A) microbial biomass carbon or (B) substrate carbon**. Linear regression statistics are given for the combined high and low tradeoff scenarios in the DEMENT model.

**Figure 4:**
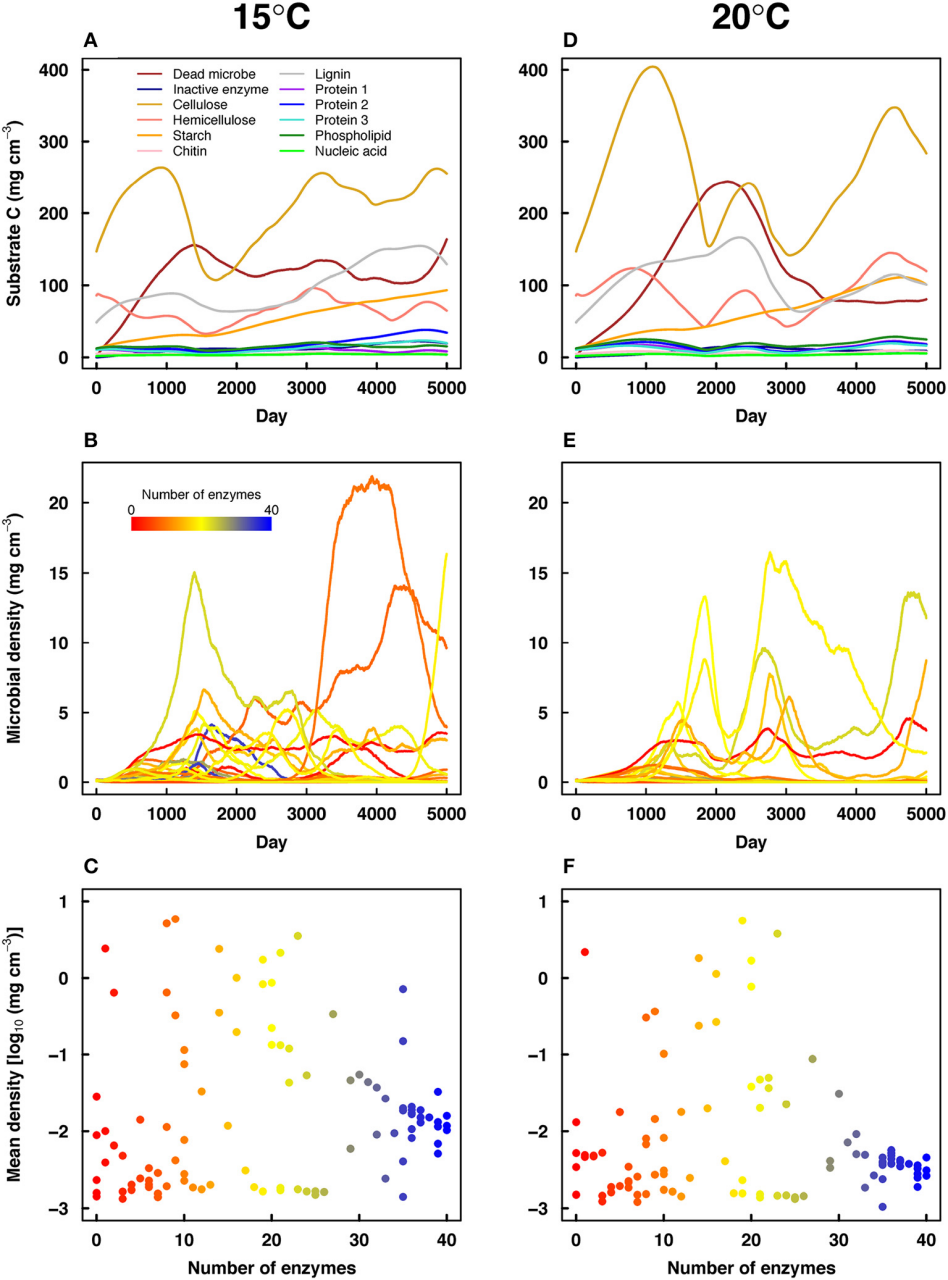
**Substrate dynamics (A,D), microbial dynamics (B,E), and mean microbial abundance vs. the number of enzymes possessed by each taxon (C,F) in a selected pair of high tradeoff DEMENT simulations at 15°C (A–C) and 20°C (D–F)**. Line colors in **(B,E)** correspond to the number of enzymes shown in **(C,F)**.

Stochastic processes in DEMENT led to variation in substrate dynamics and the degree of CUE adaptation across simulations. Runs with greater CUE adaptation had greater microbial community biomass (Figure 3A) and significantly greater gains in soil C with warming (Figure 3B). There was also variation in substrate dynamics, with substrate concentrations inversely related to their associated enzyme concentrations. For instance, starch concentrations increased in the example simulation shown in Figure 4 due to a low abundance of taxa with starch-degrading enzymes. Due to random trait assignment and population dynamics, substrate dynamics differed widely across replicate simulations.

The high tradeoff scenario showed a clear suppression of high-enzyme taxa in most runs compared to the low tradeoff scenario. High-enzyme taxa were unable to increase their biomass due to low CUE values (Figure 4). Overall, the high tradeoff scenario had significantly lower community mean intrinsic CUE, greater substrate pools, and lower average microbial biomass (*P* < 0.001, *t*-test, *n* = 40 for all three variables). With a weaker tradeoff, taxa with >30 enzyme genes often achieved high abundance and contributed to relatively lower substrate pools.

## Discussion

Previous modeling studies have shown that soil C dynamics are sensitive to changes in CUE (Allison et al., 2010; Li et al., 2014), but the physiological mechanisms underlying such changes have not been identified. The rate-yield tradeoff is an established principle in microbiology that provides a mechanistic explanation for changes in CUE (Pirt, 1965; Pfeiffer et al., 2001; Frank, 2010). The analytical model combines this mechanism with linear temperature dependence of CUE and Arrhenius uptake kinetics. The model demonstrates that microbial growth is maximized when CUE adaptation equals one-half the temperature-induced change in CUE. Microbial growth declines as the steepness of the rate-yield tradeoff increases due to lower conversion of resource uptake into biomass.

In contrast to the analytical model, DEMENT suggests a much lower potential for adaptation. Intrinsic CUE was only 0.014 mg mg^−1^ greater with 5°C warming, whereas the difference predicted by the analytical model was 0.04 mg mg^−1^. A key difference between the analytical model and DEMENT is the way growth scales with enzyme investment. The relationship is linear in the analytical model but not in DEMENT. Taxa with more enzymes do not necessarily grow faster in DEMENT because some enzymes have redundant functions. Also, the spatial structure of DEMENT means that substrate concentrations can be drawn down in the local environment of individual cells. Thus, there are diminishing returns on increasing enzyme and uptake investment. Importantly, this spatial structure allows for coexistence of taxa that fall along different points on the rate-yield tradeoff (MacLean, 2007; Bachmann et al., 2013).

At the same time, microbes in DEMENT are constrained in their ability to reduce enzyme and uptake investment. Unlike in the analytical model, enzymes in DEMENT catalyze specific reactions on distinct chemical substrates. Reducing enzyme number increases the probability of missing an essential function for a given environment. Warming selects for taxa with fewer enzymes (and higher intrinsic CUE), but taxa require a minimum number of genes to access carbon, nitrogen, and phosphorus resources. Thus, stoichiometric requirements can constrain CUE adaptation in DEMENT, consistent with previous studies on the importance of substrate stoichiometry for CUE and other microbial processes (Keiblinger et al., 2010; Sinsabaugh et al., 2013; Mooshammer et al., 2014).

Some taxa in DEMENT cooperate through consortia that reduce the number of resource acquisition genes needed by each individual taxon. Consortia are evident in Figures 4B,E as coupled oscillations in population sizes of different taxa. Similar behavior was observed in an earlier spatially-explicit model with multiple nutrients but fewer taxa (Folse and Allison, 2012). Temperature increase might be expected to favor cooperation among taxa with fewer genes and higher CUE, but this pattern is not observed in the simulations. Rather, consortia represent interdependencies among taxa, such that temperature-induced reductions in high-enzyme, low-CUE taxa also reduce the abundances of co-occurring low-enzyme, high-CUE taxa.

Another distinct feature of DEMENT is that CUE adaptation is sensitive to the strength of the rate-yield tradeoff. Weakening the tradeoff results in significantly less CUE adaptation. This pattern may be due to the greater change in enzyme investment required to achieve the same amount of CUE adaptation. For example, the average number of enzymes would have to decline by 8 in the complex model in order to achieve 50% adaptation under the low tradeoff scenario. Such a decline should be achievable in terms of average resource flux because the optimal number of enzymes starts out higher under low tradeoffs. Yet such a large decline might be selected against due to the stoichiometric constraints and microbial interactions already discussed. In DEMENT, the change in enzyme number appears to be constrained to a small value near 1 regardless of the tradeoff scenario.

DEMENT clearly shows that even if CUE does adapt, the effect on substrate degradation runs counter to previous models (Allison et al., 2010; Wieder et al., 2013). Higher levels of CUE adaptation result in greater microbial biomass pools, as expected, but greater biomass does not reduce substrate pools (Figure 3). In contrast, the change in substrate pools under warming is more positive in simulations with greater CUE adaptation. This pattern is driven by the rate-yield assumption: microbes with greater CUE have fewer enzymes, which reduces rates of substrate degradation. In previous studies, there was no mechanism or cost associated with CUE adaptation, so this feedback did not occur (Allison et al., 2010; Wieder et al., 2013).

It is difficult to know without more empirical data whether the analytical model or DEMENT is closer to reality. The analytical model requires many fewer parameters, but it lacks key features of the soil environment that are captured by DEMENT. Adding complexity such as nutrient stoichiometry, spatial structure, and functional diversity results in more potential constraints on CUE as temperature changes. Regardless of which model is closer to reality, one can conclude that additional complexity reduces the potential for CUE adaptation.

Another open question is how the DEMENT results apply to different soil environments. The current model focuses on plant litter decomposition, but the approach could be applied to other environments such as mineral soils or wetlands with adjustments to the parameters and diffusion constraints. Mineral interactions are now represented in some microbial models, albeit at larger scales than DEMENT (Wang et al., 2013; Wieder et al., 2014). DEMENT can also incorporate additional physiological traits to define microbial life history strategies in other environments (Evans and Wallenstein, 2014). The current model represents bacterial and fungal heterotrophs, but symbionts, pathogens, or autotrophs could be included by adding new trait relationships.

Although the analytical model predicts CUE adaptation, there is little evidence from DEMENT that CUE will adapt to warming through reduced investment in resource acquisition. Adaptation requires a reduction in genes essential for stoichiometric balance, microbial interactions, and resource flux. Even when CUE adaptation occurs, the rate-yield tradeoff results in lower functional potential for decomposition in DEMENT. Thus, physiological mechanisms lead to unexpected constraints on the climate response of microbial communities and their associated rates of carbon cycling. Future empirical work should focus on measuring the temperature sensitivity of CUE across ecosystems and its potential for adaptation under warming.

## Author contributions

Steven D. Allison conceived the project, designed models, analyzed data, and wrote the manuscript.

### Conflict of interest statement

The author declares that the research was conducted in the absence of any commercial or financial relationships that could be construed as a potential conflict of interest.
